# Hygienic Clothing

**Published:** 1886-02

**Authors:** L. L. McArthur


### Hygienic Clothing. *By* L. L. McArthur, M. D.

[Abstract of a paper read before the Chicago Medical Society on January 4th, 1886.]

The object of clothing should be the promotion and maintenance of good health, together with a feeling of well-being under all atmospheric conditions.

Consideration of the subject naturally subdivides itself into:

I. Materials.

II. Texture.

III. Form of clothing.

Chief among the materials used for clothing in the order of their respective merits are linen, cotton, silk, and wool; the latter being the best.

A few words as to these materials in the raw state.

Linen conducts heat better than cotton, silk or wool. It absorbs moisture and does not shrink. Cotton, also a vegetable fibre, which is hard, durable and does not shrink, has serious objections, in that it is very non-absorbent of moisture. It does not conduct heat as well as linen, but more rapidly than silk or wool.

Silk, an animal product, consisting of fine, smooth, round fibrillæ, having been in the liquid condition before leaving the body of the silkworm, possesses no central canal, and no oily coating. It is quite a good absorbent of moisture, ranking next to wool. At ordinary temperatures it always contains between 9 and 12 per centum of moisture. In its sale or purchase, account is taken of the amount of moisture in order to protect the purchaser from paying silk-prices for water.

Wool, the fleece of sheep, an animal fibre, whose function in nature has been two-fold, the protection from cold, and an aid to evaporation of cutaneous moisture, is admirably fitted as a material for clothing. It permits but slow radiation of heat, and absorbs moisture better than any other clothing-material. It does this, according to Parkes, in two ways. 1st. By interposition between the fibres. 2d, By penetration into the central canal. His experiments led him to believe its hydroscopic powers double in proportion to its weight and quadruple for surface, as compared with cotton or linen. Perfectly dried wool has the power of absorbing 50 per centum, by weight, of water. Under ordinary conditions it contains 12 to 18 per centum.

Other things being equal, then, woolen fabrics will best prevent too rapid radiation of heat, silk next, cotton third, linen fourth.

Wash-leather, buckskin, and chamois-skin need not be considered, for one or all of the following reasons: Expense, poor provision for evaporation, poor washing qualities.

As absorbers of moisture rank, respectively, wool, silk, linen and cotton.

The advantages of cotton over wool lie in its cheapness and non-shrinking qualities. If properly constructed, however, the advantageous properties of wool can be utilized, without the shrinking by using a cotton framework, into the meshes of which the loose raw fleece is worked.

That fabric will be best adapted for health, which combined with greatest porosity, possesses the least (a) conductivity, (b) greatest hydroscopic power, and (c) best shape.

By porosity is understood the freedom with which air can pass through the interstices of a fabric. Pettenkoffer's practical demonstrations with the following cloths showed that if heavy flannel be taken as permitting 100 parts of air to pass, linen permitted 60.3 or 60 per centum; lambskin 50.7 or 50.7 per centum; silk fabric, heavy, 41.4 or 41.4 per centum; glove-leather 1.5 or 1.5 per centum.

The conclusion follows that porosity does not injure the powers of preventing radiation, (it even increases it) for flannel, admittedly the warmest clothing, permits the freest circulation of air.

In consequence of the fact of a fabric's possessing great porosity, it contains in its interstices, what might be called "residual air." Whether gases possess conductivity is open to discussion, but this is certain, that such power is very small. Could we by any means envelope the body in a layer of stationary air we could reduce the heat-loss to a minimum.

A striking example of the poor conductivity of *stationary air*, is related by Dr. Kane, the Arctic explorer, who found that on perfectly still days they could withstand without suffering a temperature of —70 F., with ears and hands exposed, but the moment a breeze sprang up it became necessary to seek immediate shelter.

Although impossible to perfectly accomplish this (i. e., the surrounding of body in a stationary envelope of air), that cloth or fabric which most nearly approximates this, other things being equal, will prove itself the warmest as well as best adapted for evaporation of cutaneous moisture. Such a nearly stationary air occurs naturally in the various pelts, and although in many the integument is visible beneath, yet they can withstand the most rigorous weather. Thus Krieger's experiments with tin cylinders containing hot water with two coverings of different materials, between which an interval of 1/8 to 1/4 inch was left, proved (after subtracting the amount due for conduction) the impediment to radiation by the second layer to be, viz.: linen, 32; silk, 32; flannel, 29.

Thus showing that the stationary air, rather than the material out of which the second layer was made, was the main factor in preventing radiation.

He then experimented with single and double layers of the same material surrounding these cylinders, obtaining the following instructive results; the numbers representing the proportionate loss of heat through double to single layers, the losses through the single ones being taken as 100.

| | | |
|---|---|---|
| Double Stuff "Doppel Stoff" | Fleece-lined cotton, | 69—76 |
| Buck-skin, | " " " " " | 74—86 |
| Flannel, | " " " " " | 86 |
| Home-spun linen, | " " " " | 91 |
| Stout, extra-heavy silk, | " " " " | 94 |

From these results the conclusion is obvious that the substance and its weight are of less consequence, where *radiation* is in question, than its texture and volume. Believing that the explanation was due to the "residual air," experiments have been made with loose wadding, noting the rapidity of fall of temperature, on compressing the same wadding, when the fall was far more rapid.

Again, the loss of heat through a rabbit's fur being taken as 100, when shorn of its hair it rose to 190; and further destroying its porosity by a coating of gum-arabic, it rose to 296. (Dict. Hygiene.)

By greatest porosity best provision is made for the evaporation of perspiration, the quantity of which varies greatly under different conditions. In a day of rest the amount as determined by Seguin and Voit is 900 grams (about 1 quart). During exercise it may increase to quantities incredible, were the figures not furnished by the best of observers. For example, Dalton mentions its increase to 380 grams per hour! and Dr. Southwood Smith has seen it rise to 1,600 grams per hour during violent exercise in a heated atmosphere! Now if a clothing possess no porosity, *e. g.*, the mackintosh, and rubber clothing generally, even without exercise there would collect somewhere beneath it a quart of water, but if exercise be indulged in, the quantity may become large indeed; particularly after the atmosphere beneath has been surcharged with vapor and evaporation ceases to occur from the surface, and with it the grateful cooling process. The French Government has not permitted its introduction into its army for such obvious reasons. Of course, for a short time during a shower they may and do prove useful; but I am convinced that many have incurred most serious injury, even death, by throwing off the rubber clothing, after the inner clothing has become permeated with moisture, when the chilling, incident to the sudden increased evaporation, has resulted in some acute inflammation.

Moreover, the evaporation of the normal cutaneous moisture (with that of the lungs) requires 750 heat units or one-fifth of all the heat produced in the system (Dalton). Conservation of part of this loss, contributes an equivalent amount of force to the organism, since heat and force are interchangeable terms. *This can be done!*

Under normal conditions evaporation of perspiration occurs in the "insensible," *i. e.*, vapor-state, but change of these conditions (increased heat, and moisture in the atmosphere, increased exercise, etc.,) causes it to collect upon the integument in the visible or sensible state, and unless conducted away, may chill the body. Prevention of such condensation will avoid such dangerous and deleterious influences. The cause of condensation is a lowering of the temperature. We have simply to maintain its temperature until at a perceptible distance from the body. This can be accomplished by a layer of loose wool, such as is hereafter described. The "residual air" having been once raised to the body temperature, it remains so, and the vapor does not assume the liquid state until meeting with the chilling influences in the outer layer of cloth.

Finally, bodies passing from the gaseous to the liquid state emit the heat—latent heat—which was essential to their assuming the gaseous condition. This occurring in the case of perspiration in the cloth-interstices increases by just so much their warmth, in other words lessens the demand for heat-production.

Before leaving the subject of texture, note should be made of the importance of its being of a loose nature. However great the hydroscopic power of a material in the raw state, if it be tightly woven that power is greatly diminished, or even quite destroyed. Hence the advantage of loosely-knitted over tightly-woven goods.

Important indeed is the proper fitting of clothing. However good the materials they may then not accomplish their purpose for the following reasons :

I. By close application to the skin certain materials acting as cutaneous stimulants, maintaining an active equable circulation. Wool possesses this property most markedly; even in some delicate skins proving an irritant. A very marked increase of oily matter is excreted over those areas where oil-glands exist in greatest abundance, *i.e.*, mesial line of thorax, in front and behind ; thus improving the flexibility of the skin.

II. By fitting neatly, chambers of air heated by the body are not with every change of position of the wearer forced out, as occurs in illy-fitting clothing. Upward currents of air naturally occur, and if permitted to exist carry off large amounts of caloric. Simple attention to these two facts reduced the death-rate of the Würtemburg Army Corps, from 3.22 to 1.64, as compared with the other departments of the German Army.

The general application and advantages of such an ideal clothing to diseased conditions, it is needless for me to describe to a body of medical men ; but particular references ought to be made to rheumatism and nephritis ("kidney troubles"). To the former because best provision is made for cutaneous elimination (always acid !) so essential in that disorder, in which there is so marked a diminution in the alkalinity of the blood ; to the latter because sudden congestions are obviated in an organ already overworked, by preventing sudden chilling of the surface.

It only remains for me to call your attention to my accidentally finding such a clothing upon a patient of mine (Mr. Jaros), and the tests to which I have put it.

He described its history and manufacture as follows:

" While suffering from an attack of rheumatic sciatica in the Harz mountains, following a peasant's advice I enveloped myself in loose lamb's fleece, which he provided, and I experienced speedy relief. On reaching Berlin I consulted Chief-Councillor-of-Health, Dr. Abarbanell, who advised me to have constructed some underwear with a fleece lining. I sought a weaver and had some underwear knitted, into the meshes of which were worked, 'by hand,' during the process of knitting, layers of loose lamb's wool."

Now, gentlemen, this device was a particularly happy one, in that all the requirements of a truly hygienic wear are provided for.

Porosity, warmth, absorbent powers, and elasticity. With advice he set to work and perfected a modification of the knitting-machine which incorporated into the meshes of the cloth, loose lamb's wool. The samples presented speak for themselves as to its success. By the use of such a fabric, perspiration (unless excessive indeed) remains in the insensible state until it meets with the cooling influences externally in the cotton framework, the integument remaining dry; while the cotton back, as well as the linen shirt over it, may be " wringing wet." Exposure to cool draughts with such a suit does not chill the integument because the sudden increased evaporation occurs at a distance from the skin, and is separated from it by a layer of wool.

To test the soundness of the theory I submitted myself to a temperature of 115°F., under as nearly as possible the same atmospheric conditions, with the three chief winter suitings, and obtained the results in table below:

| | Jaros Hygienic Wear. | "Nonotuck" Silk-suiting, heavy. | Cartwright & Warner's. |
|---|---|---|---|
| Weight after | 8,020 | 7,867 | 10,840 |
| " before exposure | 7,010 grs. | 7,140 | 9,600 |
| Difference | 1,010 grs. | 727 grs. | 1,240 |
| Degree of absolute dryness of air | 61,827 | 77,32 | 69,947 |
| Temp. dry bulb therm | 115°F | 113°F | 116° |
| " wet " " | 89° | 83° | 88° |
| Sensations | Warm but not sticky; outer surface damp; skin dry where wear touches; comfortable. | Cooler than other wear; sticky; skin damp; comfortable. | Sticky; clammy; wet through; uncomfortable. |

From these experiments it is to be seen, that of all the perspiration exuded, the silk retained (by a small amount) the least; the hygienic wear the next, and the English woolen goods the most. Note, however, must be taken of two facts concerning the experiment with the silk-clothing.

1st. The temperature was 2°F. lower than when testing the hygienic wear and 8° than the English goods. Hence less perspiration was thrown out.

2d. There was a difference of 15.5° of *absolute* dryness of the atmosphere, hence evaporation took place more rapidly from the silk-goods in the dryer atmosphere. The barometer remained almost stationary during the three days of observation.

On emerging from the hot-room into one of a temperature of 70°F., an immediate chilling was felt with the silk goods; while the English gave a sensation of moisture and cold. The chilly sensation *was not experienced* with the woolen-lined hygienic-wear.

CONCLUSIONS.

1st. That the fleece-lined goods are warmest.

2d. Permit at least equal evaporation with the silk.

3d. Guard against sudden chilling of the body.

4th. Are cheaper than silk and as cheap as Cartwright & Warner's.

5th. Are particularly indicated in rheumatism and kidney-disease.

